# Discriminating Free Hand Movements Using Support Vector Machine and Recurrent Neural Network Algorithms

**DOI:** 10.3390/s22166101

**Published:** 2022-08-15

**Authors:** Christoph Reichert, Lisa Klemm, Raghava Vinaykanth Mushunuri, Avinash Kalyani, Stefanie Schreiber, Esther Kuehn, Elena Azañón

**Affiliations:** 1 Department of Behavioral Neurology, Leibniz Institute for Neurobiology, Brenneckestr. 6, 39118 Magdeburg, Germany; 2 Center for Behavioral Brain Sciences (CBBS), Universitaetsplatz 2, 39106 Magdeburg, Germany; 3 Forschungscampus STIMULATE, Otto-Hahn-Str. 2, 39106 Magdeburg, Germany; 4 Department of Neurology, University Medical Center, Leipziger Str. 44, 39120 Magdeburg, Germany; 5 Faculty of Computer Science, Otto-von-Guericke University, Universitaetsplatz 2, 39106 Magdeburg, Germany; 6 Institute for Cognitive Neurology and Dementia Research (IKND), Otto-von-Guericke University, Leipziger Str. 44, 39120 Magdeburg, Germany; 7 German Center for Neurodegenerative Diseases (DZNE), Leipziger Str. 44, 39120 Magdeburg, Germany; 8 Hertie Institute for Clinical Brain Research (HIH), Otfried Mueller-Str. 27, 72076 Tuebingen, Germany

**Keywords:** stroke, motor disorders, neurodegeneration, motor system, quantification, data glove

## Abstract

Decoding natural hand movements is of interest for human–computer interaction and may constitute a helpful tool in the diagnosis of motor diseases and rehabilitation monitoring. However, the accurate measurement of complex hand movements and the decoding of dynamic movement data remains challenging. Here, we introduce two algorithms, one based on support vector machine (SVM) classification combined with dynamic time warping, and the other based on a long short-term memory (LSTM) neural network, which were designed to discriminate small differences in defined sequences of hand movements. We recorded hand movement data from 17 younger and 17 older adults using an exoskeletal data glove while they were performing six different movement tasks. Accuracy rates in decoding the different movement types were similarly high for SVM and LSTM in across-subject classification, but, for within-subject classification, SVM outperformed LSTM. The SVM-based approach, therefore, appears particularly promising for the development of movement decoding tools, in particular if the goal is to generalize across age groups, for example for detecting specific motor disorders or tracking their progress over time.

## 1. Introduction

The decoding of natural hand movements may constitute a helpful tool in the diagnosis and tracking of motor diseases. Motor diseases in which the hand is affected include, for example, stroke, Parkinson’s disease (PD), and amyotrophic lateral sclerosis (ALS), where there is a general lack of quantification of behavioral disease characteristics [1]. A major challenge in decoding natural hand movements and their potential change in conditions of disease is that the movements are both dynamic and idiosyncratic. Alignment of common temporally matching features is, therefore, difficult, because individuals show slightly different hand movement patterns and hand dynamics. Furthermore, hand geometry, as well as age, might have an influence on the recorded characteristics, which could make generalization across different individuals difficult. Thus, person-independent detection of underlying characteristic hand movement patterns is challenging. On the other hand, individual differences could provide meaningful information on underlying and undetected disorders, as well as developmental changes through the individual lifespan, and may provide new ways to study the neural mechanisms that underlie natural hand movements in humans. In this respect, the challenge is to implement an algorithm that disregards idiosyncratic characteristics, such as hand size and other individual attributes, such as age, but detects small differences in the movement pattern.

Another unsolved question concerns how far decoding algorithms can detect common hand movement features across different age groups. Individual age is a strong predictor of motor cortex architecture and sensorimotor behavioral decline, and massively influences a range of sensory and motor tasks [2,3]. However, to use the decoding of hand kinematic features in a clinical or applied context, it is of critical importance to be able to generate models that are independent of individual hand features, as well as individual age. Only if such generalization is successful can algorithms be used to identify and track specific disease-related features over time and in clinical contexts.

Several devices exist that are suitable for tracking movements of the hand, fingers, or finger segments. These include visual-based tracking devices, data gloves with bending sensors, exoskeleton gloves, and inertial measurement units (IMUs). Finger tracking using camera-based data is a technique that has the great advantage that it does not require contact with any device. In early work, hand models were introduced to improve gesture detection [4,5]. In recent years the development of image-based hand tracking has made tremendous progress. For example, the Leap Motion Controller (UltraLeap, Mountain View, CA, USA) is a commercially available stereo camera which comes with developer software implementing a skeletal hand model [6]. Machine learning has led to the development of robust open-source methods that can track hand positions using 2D images, e.g., DeepLabCut (github.com/DeepLabCut, accessed on 7 August 2022) [7], InterHand2.6M (github.com/facebookresearch/InterHand2.6M, accessed on 7 August 2022) and MediaPipe (google.github.io/mediapipe, accessed on 7 August 2022). The disadvantage of camera-based methods for continuous finger tracking, especially when the manipulation of objects is involved, is that occlusions would lead to missing or inaccurate data of the occluded tracking points and determination of accurate finger joint angles would be challenging. An overview of additional commercial devices for hand movement tracking is given by Caeiro-Rodríguez et al. [8]. Most of these devices are designed for use in virtual reality (VR) environments, as well as for human–computer interaction, and thus are not necessarily suited for accurate recording of complex hand kinematics in non-VR applications. Nevertheless, several applications of such devices, other than VR control, have been proposed, such as sign language recognition [9,10,11], hand function evaluation [12], surgical skill analysis [13], teaching robots with grasp movements [14], and hand rehabilitation [15,16]. In the present study, we used a recently developed exoskeleton glove (SenseGlove DK1.3, Delft, The Netherlands), which measures angular position of its joints, and which is suitable for accurate tracking of single fingers during complex hand movements.

Here, we introduce two approaches that we expected to be suitable to decode a dynamic but well-defined sequence of single-finger gripping movements. In the first approach, we employed dynamic time warping (DTW) and support vector machine (SVM) classification to recognize types of hand movement sequences. Both DTW and SVM are established algorithms that can be sophisticatedly combined. In the second approach, we employed long short-term memory (LSTM) units. LSTM units are suited to appropriately analyze long time lags and should therefore be suitable to decode hand movements with varying time length from trial to trial and across individuals. LSTM was first introduced to handle the problem of vanishing or exploding gradients caused by vanilla recurrent neural network (RNN) models in solving sequencing model tasks [17]. Since the inception of LSTMs, they have gained popularity, not only for appropriately analyzing sequences, but also in time series [18], dynamic modeling, speech recognition [19] and other complex decoding tasks [20,21].

By applying these algorithms to the analyses of highly similar hand movements in two age groups of individuals, we show that our approaches reveal reliable results in the decoding of hand movement types and are robust in terms of generalizability across individuals and age groups. With our analyses, we demonstrate that SVM combined with DTW is preferable compared to LSTM because it is more accurate with small datasets and comparably accurate with large datasets, while it is computationally much more efficient. The decoding of highly similar hand movements in healthy younger and older individuals serves as a proof-of-concept study and suggests a promising method for developing diagnostic tools for motor disorders, or to develop algorithms to successfully track disease progress or healing success at home.

## 2. Materials and Methods

### 2.1. Participants

We recruited two groups of healthy participants, older adults (N = 17; 10 female and 7 male, mean age 72.2 ± 4.8 years ranging from 62 to 80 years) and younger adults (N = 17; 10 female and 7 male, mean age 27.0 ± 4.3 years, ranging from 22 to 37 years). All participants reported intact upper limb motor abilities and intact tactile perception of the hand and were naïve regarding the purpose of the study. They were paid for their participation and gave written informed consent. The study was conducted in accordance with the principles of the Declaration of Helsinki and was approved by the Otto-von-Guericke University Magdeburg Research Ethics Committee (Germany).

### 2.2. Experimental Approach

**Figure 1:**
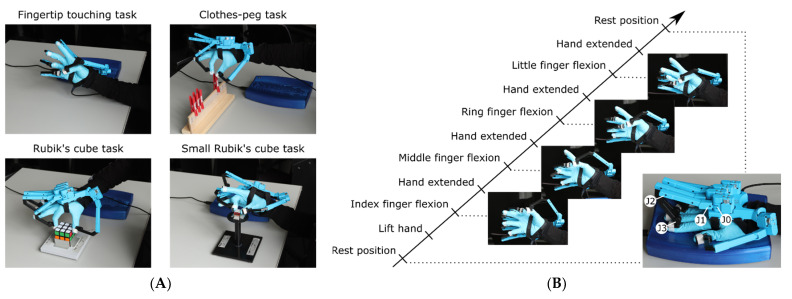
Movement tasks. (**A**) Four different tasks were performed requiring precision grips of the index, middle, ring and little fingers. Note that the Rubik’s cube and small Rubik’s cube tasks were performed twice, i.e., clockwise and counterclockwise (see also Supplementary Video S1). (**B**) One trial comprised a sequence of grips of each of these fingers performed self-paced (except the fingertip touching whose movements were triggered every 2 s by an acoustic signal). In the image showing the rest position, the exoskeleton data glove’s joints of the index finger are exemplarily annotated (abduction sensor J0 and finger flexion sensors J1–J3).

Participants were asked to perform sequences of predefined hand movements using their dominant right hand while wearing an exoskeleton data glove (see Figure 1A and Supplementary Video S1 for an overview of all movements). They performed six different sequences of hand movements, all representing a different form of pinch-grip: a fingertip touching task, a clothes-peg task, two forms of a Rubik’s cube task, and two forms of a small Rubik’s cube task. In the fingertip touching task, participants were asked to touch the tip of the thumb (D1) with each of the fingertips in consecutive order, i.e., index finger (D2), middle finger (D3), ring finger (D4), little finger (D5), as shown in Figure 1B. After each fingertip contact, the five fingers were extended as much as possible by the participants. In the clothes-peg task, participants were required to pick up single clothes pegs with two fingers (D1 + D2, D1 + D3, D1 + D4, D1 + D5) from one specified position on a board and place them on another specified position on the same board. The exact location of each clothes peg was marked on the right and left sides of the board so that participants were aware of the start and ending locations of the movement. The direction of the hand movement while holding the clothes peg, from right to left or vice versa, switched from sequence to sequence.

In the two Rubik’s cube tasks, participants were asked to rotate a 5.7 by 5.7 cm Rubik’s cube, mounted on a support, by 90°, with the palm directed downward and above the cube (see Figure 1A). Again, as for the other tasks, the rotation was made in sequence using D1 together with D2, D3, D4, D5 at a time. This task was performed clockwise (cw) and counterclockwise (ccw), mimicking the closing and opening of a jar.

Finally, participants were asked to perform the same task again but with a smaller Rubik’s cube (2.1 by 2.1 cm), here with the hand operating from the right side instead of from above the cube as in the previous Rubik’s cube task. Cw and ccw movements were performed again, this time mimicking the closing and opening of a bottle.

Participants performed the fingertip touching task at a given pace of 2 s where flexion and extension was triggered by an acoustic click cue which was presented every second. In the rest of the tasks, participants performed the movements at their own pace.

After execution of one sequence of four finger movements (D2 to D5), the hand was placed flat and static on an in-house resting pad which detected the placement of the hand. The next sequence started when the hand released the resting pad, sending a trigger signal to the recording computer. See Supplementary Materials Video S1 for a video showing all different movement types.

The above-outlined tasks were chosen to be fast and easy to instruct, to involve everyday objects, and to involve different muscle groups of the hand, making them potentially suitable for clinical application.

The experiment consisted of 300 trials divided into five runs. Each trial consisted of the four sequenced finger movements. Each run consisted of six blocks, each consisting of 10 repetitions of a given movement task, which was identical across participants, in the following order: clothes-peg task, fingertip touching, Rubik’s cube cw, Rubik’s cube ccw, small Rubik’s cube cw, and small Rubik’s cube ccw, resulting in a total of 60 trials per run. At the start of each block, a practice trial was included to familiarize participants with the required movement. Familiarization trials were not analyzed. Before the experiment started, we recorded five calibration postures for one second each, including a flat hand and a fist, to assess the minimum and maximum angles recordable at each sensor with respect to the individual montage of the exoskeleton glove. A recording session took about 2.5 h per participant, including calibration.

We took a photograph of each participant’s hand which was placed on a reference surface. The photographs were used to determine the hand geometry of each participant at 22 reference points. We used the total length of the hand, measured as the distance from the wrist to the most distal point of the middle finger, as a variable to perform analyses on hand size as described below.

### 2.3. Data Recording and Preprocessing

We recorded the movement of the fingers using an exoskeleton data glove (SenseGlove DK1.3; senseglove.com, see Figure 1B), which was equipped with four rotation sensors per finger (one abduction sensor, J0, and three finger flexion sensors, J1–J3, see Figure 1B) and a 9-DOF inertial measurement unit (IMU).

The raw sensor data were streamed to MATLAB (Release 2019a, The MathWorks, Inc., Natick, MA, USA) running on an HP ZBook Intel i7-8565U for recording and further processing. In our analysis, we involved 15 flexion sensors (J1–J3 of D1–D5) to investigate the degree of single finger flexion. The rotation sensors of the exoskeleton represent the joint angle of finger joints only indirectly. The transformation from device angles to finger joint angles is not trivial and depends on many variables, such as individual hand geometry and precise placement of the device. To circumvent this challenge, we first normalized the data such that the minimum value of a sensor as obtained during calibration postures was corrected to zero (flat hand) and the maximum value was corrected to one (full flexion of all fingers). Second, we analyzed movement data, i.e., the temporal derivative of the glove’s joint angles, which is largely independent of hand geometry, in contrast to posture data.

The glove data were sampled at 100 Hz. We applied a moving average filter of 100 ms width to remove noise. Since natural hand movements are of low frequency, we down-sampled the signals to 16 Hz to reduce the feature space for the learning algorithm and the associated computational costs.

### 2.4. Decoding Algorithms

Each preprocessed time series of angular velocity, reflecting movement sequences of fingers D2 to D5, was considered a trial, and we aimed at classifying this trial according to the movement type. To this end, we made use of and compared two different decoding algorithms. The first algorithm uses support vector machine (SVM) classification which requires the data to be represented in a unique feature space. To transform the dynamic signals into a unique feature space, we used dynamic time warping (DTW) to adjust the time series. The second algorithm makes use of an LSTM recurrent neural network which is suited to directly learn temporal dependencies in time series of varying lengths.

We applied within-subject analyses using the data of individual participants and across-subject analyses aiming at predicting the movement type in an unseen participant. In within-subject analyses, we applied leave-one-run-out cross-validation, i.e., all trials corresponding to one run were left out for classification while the classifier was trained on the remaining runs. In across-subject analyses, we performed leave-one-subject-out cross-validation, i.e., all trials of an individual participant were left out for classification while the classifier was trained only on trials of all other participants. This latter analysis tests for the generalization ability of the approach since no data of the to-be-classified participant is involved in the learning phase. We also applied two-fold cross-validation in which we trained and tested on data of two different subsets of participants to demonstrate that the task prediction also generalizes across different hand sizes and age groups. We performed the two-fold cross-validation using three different approaches. In the first approach, we selected two subsets where both hand size and age were matched (matched split). In the second approach, one group of participants was selected by hand size, the 17 smallest in one group and the 17 largest in the other group (hand size-grouped split). Finally, in the third approach, the two groups were selected by age, i.e., 17 younger adults and 17 elderly adults (age-grouped split). Note that, by splitting the data into only two subsets, the training set size is decreased, and the testing set size is increased compared to the leave-one-subject-out approach described above.

We calculated the decoding accuracy (DA) achieved with each cross-validation to evaluate our results. We determined a significance level by permuting the labeling of movement tasks, i.e., we assigned the classifier labels corresponding to each task randomly to each trial. We applied the analysis as with the original labeling and repeated the randomization 1000 times with randomly labeled data. This resulted in 1000 DAs. We determined the 95% confidence interval (CI) of this DA distribution by calculating the 2.5th and 97.5th percentile and setting the upper CI limit as the significance threshold.

#### 2.4.1. Support Vector Machine Combined with Dynamic Time Warping

Single movements were executed at different time points, with differences in accelerations, decelerations, and durations. This resulted in different lengths of the entire movement sequence across repetitions and participants. To perform support vector machine-based classification, we first needed to align trials in time. To this aim, we applied dynamic time warping (DTW) as implemented in MATLAB’s Signal Processing Toolbox. While DTW calculates a similarity measure between signals, it generates a warping path, which we used to realign the time series. To sample the time series to a constant length, we first determined the most representative time series template across tasks and participants. Time series were then sampled to the same length as the template and realigned to have a minimum sum of Euclidean distance to the template using DTW.

**Figure 2:**
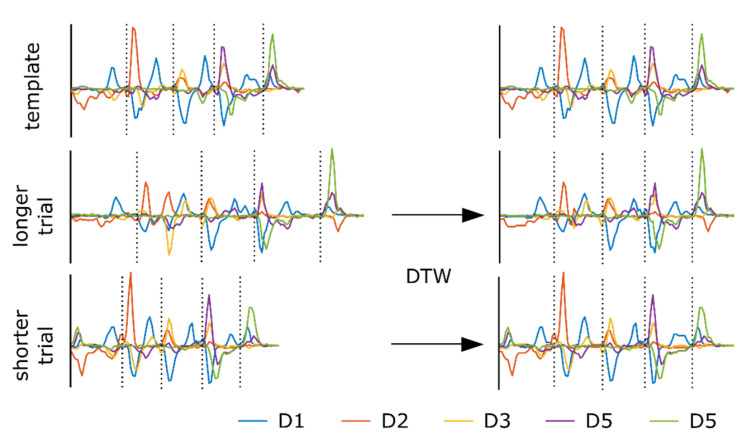
Signal synchronization utilizing dynamic time warping (DTW). For simplicity, we show only J2 angular velocities of fingers D1–D5 for two exemplary trials of the small Rubik’s cube task (cw), one longer and one shorter than the template trial. The resulting time series are sampled to the length of the template and have a minimum sum of Euclidean distance to the template. As the vertical dotted lines in each plot show, the different segments of the signals are asynchronous before DTW and are synchronous after DTW.

Let $X_{i}\in {\mathbb{R}}^{t\times c}$ be a set of time series representing a trial where t is the number of samples and c is the number of channels. To find a representative template, we performed DTW between trial i and trial $i+\frac{n}{2}\;(i=1,\ldots ,\frac{n}{2}-1$) where n is the number of all trials in the training set. This represents only a small subset of all available pairwise combinations but, to reduce computational costs, we considered it sufficient to find a representative time series that was not an outlier. For each pairing, we calculated a score $s_{i}=\rho_{i}\left(1-d_{i}\right)$ where $\rho_{i}$ is the correlation coefficient calculated between the ith aligned trial pairing and $d_{i}$ is the sum of Euclidean distances as revealed by the DTW algorithm but normalized such that $\underset{i}{\max}d_{i}=1$. We selected the first trial of the pair that provided the maximum score $s_{i}$ as a template T. Next, we calculated the DTW between each trial $X_{i}$ and the selected template trial T (see Figure 2). The warping paths that minimize the sum of Euclidean distance between the signals include repetitions of time points in either time series. To resample the time series $X_{i}$ to the length of the template time series T, data points that resulted from consecutive repetitions in T were averaged in $X_{i}$. This approach synchronized the four single movements of a trial and transferred the time points to a feature space of constant size which is required for training an SVM. Here, we applied linear SVM classification in a one-vs-rest framework to discriminate between different classes. For SVM training, no parameter optimization was performed but the default box constraint parameter C, as suggested by Joachims [22], and used in the SVM*^light^* implementation (cs.cornell.edu/people/tj/svm_light, accessed on 7 August 2022), was used:

(1) $$C=n{\left(\overset{n}{\underset{i}{\sum }}x_{i}x_{i}\right)}^{-1}$$

where $x_{i}$ is the vector form of the feature matrix $X_{i}$ and n is the number of training samples.

#### 2.4.2. Long Short-Term Memory Neural Network

In contrast to support vector machines, neural networks involving LSTM units can analyze dynamic data without padding or altering the length. They have proved to be very effective in learning long-term dependencies in the input sequences. An LSTM unit is composed of one or several self-connected memory cells, an input gate, an output gate and a forget gate. The gates perform multiplicative operations to control the flow of information. The input gate regulates the amount of cell state information to be involved in the network. Similarly, the forget gate specifies information to forget and information to be kept in the cell state. Finally, the output gate specifies the information to be sent to the next hidden state. See Graves [23] for a comprehensive background review of LSTM units.

**Figure 3:**
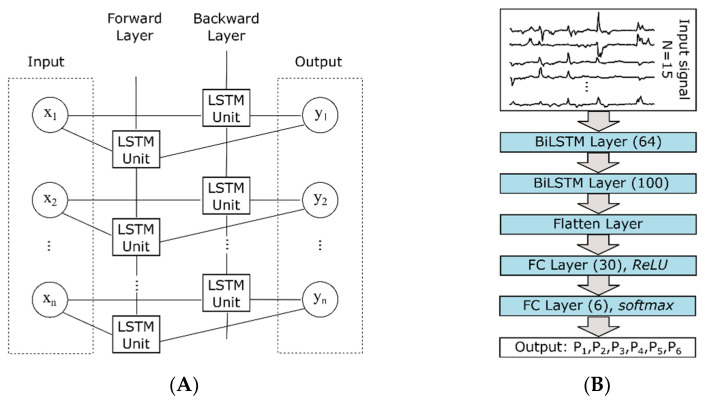
The architecture of the neural network. (**A**) shows the model of a BiLSTM layer with n hidden units. (**B**) shows the layers that were used to train the RNN. Numbers in parentheses indicate the number of hidden units.

In this study, we have employed two stacked bi-directional LSTM (Bi-LSTM) layers, followed by one hidden layer with 30 neurons, followed by a ReLU (rectified linear unit) activation function. The final layer contained six neurons with *softmax* as an activation function to permit the classification of six different classes. Figure 3B depicts the block diagram of the architecture used. Bi-LSTM was employed since it contains two hidden states at each point in time, one hidden state to process information in a forward direction (from past to future), and another in a backward direction (from future to past), unlike common LSTM. Its advantage is that at any instance it preserves information from both past and future. Stacked Bi-LSTM layers were followed by layer normalization and a ReLU block, which encoded the dynamic hand movement data as follows: Let $X\in R^{N\times t}$ be the dynamically time-varying data of N time series. B denotes the stacked Bi-LSTM unit with layer normalization and ReLU blocks. The output vector E is defined as

(2) $$E=B\left(X\right)$$

and is passed to the decoder network, which contains a series of hidden layers followed by ReLU activations. The information from the encoder is decoded using the hidden layers and the probabilities of the hand movement types are predicted using the final classification layer. The hidden layers H assign a probability P to each of the movement types. Then the final classification output y is obtained using the argmax function:

(3) $$P=H\left(E\right)$$

(4) $$y=argmax\left(P\right)$$

We implemented the algorithm using MATLAB R2021a (Deep Learning Toolbox), and the GPU of an NVIDIA GeForce RTX 3060 Ti was employed for training. The models were trained for 64 epochs with a mini-batch size of 16. Cross-entropy loss was used as a loss function and Adam optimizer was used for optimization of the loss function with a learning rate of 0.001. We performed model optimization by a grid search approach varying the parameters: number of epochs, mini-batch-size and number of hidden units. This approach resulted in the optimal parameter set as reported above.

In order to test whether DTW would improve the performance of LSTM, we also performed the analyses using time-warped time series as we did with the SVM approach.

## 3. Results

We tested whether the SVM and LSTM classifiers were able to predict the six different movement types within the same participants (within-subject approach) and across different participants (across-subject approach). Our analyses revealed that the SVM classifier predicted the six different movement types on a single trial basis with high reliability, achieving a within-subject average DA of 99.4% (SE: 0.1) and an across-subject DA of 96.5% (SE: 0.9). In contrast, LSTM classification resulted in a lower within-subject DA of 86.4% (SE: 1.9) on average, but the across-subject DA was similar to that of the SVM classification (96.5% (SE: 0.9)). The sensitivity of single movement tasks is shown in Table 1. Although LSTM is assumed to learn dependencies in dynamic time series, we also combined DTW with LSTM and found that within-subject DA (94.9% (SE: 0.6)) was significantly higher compared to LSTM alone but statistically significantly lower compared to SVM. In contrast, across-subject analysis achieved no statistically different DA (95.9% (SE: 0.7)), neither compared to LSTM alone nor to SVM.

Permutation tests revealed a chance level DA of 16.6% (CI: 15.9–17.4%) for SVM classification and a chance level DA of 16.2% (CI:15.7–16.7%) for LSTM classification, which are close to the theoretical guessing level of a six-class problem (16.7%) and demonstrates that the classifiers were not biased. The relatively high DA of the across-subject classification suggests that the classifiers generalize across different participants.

**Figure 4:**
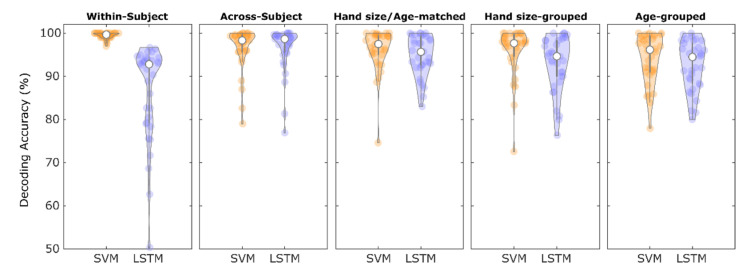
Classifier performance in differently grouped subsets to be left out in the cross-validation (CV). In within-subject CV, SVM performs considerably better than LSTM. Compared to across-subject analysis (leave-one-subject-out CV), both SVM and LSTM classification results are only slightly affected by a smaller amount of training when using two-fold CV (regardless of the grouping characteristics, i.e., matched hand size or age). In addition, grouping participants in two hand size groups and two age groups revealed similar DAs compared to two hand size/age-matched groups using the same amount of training data. Note that the chance level is at 16.6%.

We then investigated the generalizability of our approaches using a two-fold CV considering three cases: (I) matched in age and hand size (matched split), (II) split according to small and large hands (hand size-grouped), and (III) split into young and elderly (age-grouped). The DA of the CV using the matched split decreased only slightly (SVM: 96.2% (SE: 0.8), LSTM: 94.9% (SE: 0.8)), showing that training on 17 participants already led to highly reliable accuracy. Importantly, compared to the matched split, the hand size-grouped split (SVM: 95.8% (SE: 1.0), LSTM: 93.1% (SE: 1.1)) and the age-grouped split (SVM: 94.3% (SE: 1.0), LSTM: 92.6% (SE: 1.0)) did not lead to significantly different DAs as obtained with a Wilcoxon signed-rank test (Bonferroni corrected *p*-values >0.05). This indicates that the classifiers were independent of hand geometry and age-related differences in movement behavior. More precisely, despite the fact that age-related changes in finger movements have been reported using similar movements as used here (e.g., [24]), the classifiers can detect movements of older adults, although only data from younger adults were used for classifier training and vice versa. The results of all CVs are shown in Figure 4.

## 4. Discussion

There is ongoing development of tools, such as data gloves, that allow the capture of dynamic movements in real life. However, there has been limited information to date on how to analyze and decode such data, in particular when the aim is to generalize information across individuals. We introduce here two different approaches to discriminate dynamic sequences of finger grip movements in younger and older adults, that were tracked using an exoskeleton data glove. The six different finger movements involved different everyday movements required when opening a jar or a bottle, for example. The movements were reliably decoded by both algorithms and showed high generalizability, not only within participants, but also across participants and across different groups split by hand size and age. Accuracy rates in decoding the different movement types were similarly high for SVM and LSTM in across-subject classification, but, for within-subject classification, SVM outperformed LSTM. The SVM-based approach, therefore, appears particularly promising for the development of tools that make use of decoding hand movements, for example, for detecting specific motor disorders or tracking their progress over time.

First, we used SVM classification as a state-of-the-art classifier method designed to perform classification tasks in high-dimensional feature space. For this classification, it was necessary to adjust the time series data to the same number of samples such that the movement events were aligned in time. To achieve this, we applied the DTW algorithm, which has been successfully used in many applications, such as driving pattern recognition [25], gesture recognition [26], and aligning motor neural activity [27]. Second, we used an LSTM neural network, where the LSTM architecture was designed to make predictions directly from dynamic time series and, therefore, DTW was not required. Remarkably, both approaches, i.e., SVM and LSTM, decoded the movement sequences with comparable accuracy when the models were trained on data of different participants and tested on data of unseen participants. In contrast, SVM showed superior performance when data within single participants were classified. Furthermore, the DA of LSTM showed a greater decrease than that of SVM when only half of the participants were involved in teaching the models. These results are consistent with the assumption that LSTM, as a deep neural network algorithm, has its strength when trained with higher amounts of data [28], whereas SVM is known to perform well even when the number of features is much higher than the number of training samples [29]. We also compared the effect of performing DTW before training the LSTM model and found that LSTM benefitted from DTW only in within-subject analyses, i.e., when the number of training samples was low. Given that LSTM requires a great deal of computation time for model training compared to SVM, and SVM performs better with small training sets, we consider the approach of combining DTW and linear SVM to be more suitable for the proposed classification task and for future use in experimental or clinical settings where similar amounts of data and participants are used. Another argument for using SVMs is that they do not necessarily require model optimization, as is common with deep neural networks. Here, we performed a heuristic grid search approach to optimize the LSTM model which did not guarantee that the optimal model was found [30]. However, it is also likely that SVM parameters were not optimal.

The dynamic time warping approach has the advantage that delayed signals are synchronized. After that, distinctions in spatial features of time-matched movement trajectories can be detected by the decoder. However, time-warping also implies that temporal differences in movements, which might be characteristic of the intended movement classes, are cancelled out between trials. In contrast, temporal variations across fingers would not be removed. This disadvantage should be considered if such differences are key features in the signals to be classified, for example, when the duration of a movement is the key feature.

Age-related changes in hand kinematics can be remarkable. They include more rigid and slower movements, as well as reduced flexibility and precision in movement execution, in older compared to younger adults [31]. In addition, there are differences in peripheral factors, such as hand flexibility, tactile sensitivity, and skin friction that usually result in a different pattern of hand movements in older compared to younger adults [24]. It was, therefore, an open question whether the classifiers used here would be able to generate a model that would be detected independent of the obvious differences in hand kinematic features that exist between younger and older adults. If this were successful, this would imply that future approaches could use our proposed algorithms to detect disease-related changes independent of the individual age of the participant. Here, we show that the proposed algorithms generalized across different hand sizes and age groups when the classifiers were trained to discriminate different hand movement types. To detect disease-related changes in specific muscle groups, one would train the classifier to detect differences in healthy controls and patients using only one movement type performed by both groups. We hypothesize that the generalization across age groups and hand sizes would be similar in this setting.

Taken together, the algorithms presented here were evaluated on a set of similar movement sequences as a proof-of-concept study. In some motor disorders, small differences in the ability to perform natural hand movements might constitute a crucial marker for a more or less severe disorder, and the ability to track motor abilities over time may constitute a new way of using digital technology for tracking disease or rehabilitation progress. Given that such differences are difficult to detect by subjective observation, as is mostly done in clinical settings, a technical acquisition and algorithmic differentiation of hand movements could also support an earlier diagnosis of motor disorders. Future work may build on the presented algorithms to discriminate those characteristic differences and develop a workflow as a diagnostic tool.

## Figures and Tables

**Table 1:** Sensitivity of single classes.

Movement Task	Within-Subject Average DA [%] (SE)		Across-Subject DA [%] (SE)	
	SVM	LSTM	SVM	LSTM
fingertip touching	99.9 (0.1)	91.3 (2.5)	99.2 (0.4)	99.3 (0.3)
clothes-peg	99.9 (0.1)	92.5 (1.8)	98.1 (0.7)	99.1 (0.3)
Rubik’s cube cw	98.6 (0.6)	83.7 (3.3)	96.5 (2.2)	96.5 (1.6)
Rubik’s cube ccw	98.4 (0.5)	83.8 (2.5)	95.0 (2.3)	93.8 (2.1)
small Rubik’s cube cw	99.9 (0.1)	79.2 (2.5)	94.1 (2.9)	96.1 (2.8)
small Rubik’s cube ccw	99.8 (0.1)	88.2 (2.1)	96.2 (1.6)	94.2 (2.3)

## Data Availability

The data presented in this study are openly available in the open science repository for research data and publications of the Otto-von-Guericke University at 10.24352/UB.OVGU-2022-080.

## Supplementary Materials

The following supporting information can be downloaded at: https://www.mdpi.com/article/10.3390/s22166101/s1, Video S1: Movement tasks.
